# Efeitos do Óleo de Copaíba em Marcadores Periféricos de Estresse Oxidativo em um Modelo de *Cor Pulmonale* em Ratos

**DOI:** 10.36660/abc.20200929

**Published:** 2021-09-16

**Authors:** Cristina Campos, Patrick Turck, Angela Maria Vicente Tavares, Giana Corssac, Denise Lacerda, Alex Araujo, Susana Llesuy, Adriane Bello Klein

**Affiliations:** 1 Universidade Federal do Rio Grande do Sul Porto Alegre RS Brasil Universidade Federal do Rio Grande do Sul, Porto Alegre, RS – Brasil; 2 Hospital Italiano de Buenos Aires Buenos Aires Argentina Hospital Italiano de Buenos Aires, Buenos Aires – Argentina

**Keywords:** Doença Cardiopulmonar, Monocrotalina, Ratos, Estresse Oxidativo, Fabaceae, Fitoterapia, Hipertrofia Ventricular Direita, Óleo de Copaiba

## Abstract

**Fundamento:**

Até o presente momento, os efeitos sistêmicos do óleo de copaíba jamais foram documentados no *Cor pulmonale* induzido por monocrotalina.

**Objetivos:**

Investigar os efeitos do óleo de copaíba nos marcadores periféricos de stress oxidativo em ratos com *Cor pulmonale.*

**Métodos:**

Ratos Wistar machos (170±20g, n=7/grupo) foram divididos em quatro grupos: controle (CO), monocrotalina (MCT), óleo de copaíba (O), e monocrotalina + óleo de copaíba (MCT-O). Foi administrada a MCT (60 mg/kg i.p.) e, depois de uma semana, foi iniciado o tratamento com óleo de copaíba (400 mg/kg/day-gavagem-14 dias). Foi realizado o ecocardiograma e, depois disso, foi coletado sangue do tronco para a realização de avaliações de stress oxidativo. Análise estatística: ANOVA de duas vias com teste Student-Newman-Keuls post hoc. P-valores <0,05 foram considerados significativos.

**Resultados:**

O óleo de copaíba reduziu a resistência vascular pulmonar e a hipertrofia do ventrículo direito (VD) hipertrofia (Índice de Fulton (mg/mg)): MCT-O= 0,39±0,03; MCT= 0,49±0,01), e função sistólica melhorada (fração de encurtamento do VD, %) no grupo MCT-O (17,8±8,2) em comparação com o grupo de MCT (9,4±3,1; p<0,05). Além disso, no grupo MCT-O, espécies reativas do oxigênio e os níveis de carbonila foram reduzidos, e os parâmetros antioxidantes aumentaram no sangue periférico (p <0,05).

**Conclusões:**

Os resultados deste estudo sugerem que o óleo de copaíba tem um efeito antioxidante sistêmico interessante, que se reflete na melhoria da função e na morfometria do VD nesse modelo de *Cor pulmonale* . A atenuação do *Cor pulmonale* promovida pelo óleo de copaíba coincidiu com uma redução no stress oxidativo sistêmico.

## Introdução

A Floresta Amazônica poderia ser considerada um laboratório natural, por tem uma ampla diversidade de plantas com propriedades medicinais. A maioria dessas plantas ainda não foram estudadas, como é o caso da copaíba.^1^ A copaíba é uma árvore grande que cresce abundantemente na região norte do Brasil. Desde o século XVI, o óleo de copaíba tem sido usado pelos indígenas nativos do país para tratar várias doenças. Esses usos tradicionais motivaram alguns pesquisadores a estudarem esse óleo.^2^

De acordo com alguns relatos, o óleo de copaíba apresenta propriedades antioxidantes e antilipoperoxidativas.^3 , 4^ As propriedades antioxidantes do óleo de copaíba poderiam ser muito úteis no tratamento de algumas doenças cardiovasculares associadas ao stress oxidativo. Entretanto, há apenas dois estudos na literatura demonstrando os efeitos benéficos do óleo de copaíba em doenças cardiovasculares, como a hipertensão arterial pulmonar (HAP).^5 , 6^

A HAP é uma doença crônica e fatal que está associada a aumentos progressivos da pressão e da resistência vascular pulmonar. Essas alterações prejudicam o desempenho do ventrículo direito (VD) e resultam em insuficiência do VD, e, em última instância, em morte.^7^ Para se estudar os mecanismos fisiopatológicos envolvidos da disfunção do VD e no desenvolvimento da HAP, foi utilizado o modelo de monocrotalina (MCT).^8^ O metabólito ativo da MCT causa danos ao endotélio pulmonar, levando à HAP.^9^

O modelo MCT mimetiza aspectos da HAP humana, incluindo o *Cor pulmonale,* que é um termo usado para descrever a hipertrofia patológica do VD induzida por disfunção pulmonar.^10^ Na verdade, vários estudos em modelos animais e pacientes implicam o stress oxidativo no desenvolvimento do *Cor pulmonale* e da HAP.^11 - 13^ O stress oxidativo pode causar danos às células endoteliais^14^ além de contribuir para a disfunção e insuficiência do VD.^11^ Entretanto, nenhum estudo explorou o impacto da HAP nos marcadores de stress oxidativo no sangue periférico, ao analisar os efeitos do óleo de copaíba. Relatou-se que o stress oxidativo medido no sangue de pacientes com doença neurodegenerativa poderia representar um reflexo do dano oxidativo cerebral nesses pacientes.^15^ Dessa forma, a avaliação dos marcadores periféricos de stress oxidativo poderia ter uma aplicabilidade clínica, já que a obtenção de uma amostra de sangue representa um procedimento minimamente invasivo.^16^ Essa abordagem poderia ser útil em caso de doenças cardiopulmonares, tais como a HAP, para monitorar o avanço da doença, além da necessidade e da eficácia da terapia antioxidante, como o óleo de copaíba.

Portanto, o objetivo deste estudo foi investigar se os marcadores periféricos de stress oxidativo refletem as alterações estruturais e funcionais promovidas no VD pela HAP e os efeitos do óleo de copaíba sobre esses marcadores.

## Métodos

### Animais, indução do Cor pulmonale e grupos:

Todos os procedimentos foram aprovados pelo Comitê de Ética Animal institucional (número de protocolo: 31765). No total, foram estudados 28 ratos Wistar machos (pesando 170±20g) do Centro de Reprodução e Experimentação de Animais de Laboratório (CREAL) da Universidade Federal do Rio Grande do Sul. Eles foram mantidos em uma temperatura de 20-22°C, com um fotoperíodo de 12:12h escuro/claro. Todos os animais tinham acesso ad libitum a ração para ratos comum e água, e os experimentos foram realizados de acordo com o Guia para o cuidado e uso de animais de laboratório) (Departamento de Serviços de Saúde e Humanos, Publicação NIH nº 86-23), e com diretrizes institucionais.

O número de animais por grupo foi estimado com base em estudos anteriores de nosso grupo de pequisa,^6 , 7^ considerando a diferença mínima entre os grupos de dois desvios padrão, uma probabilidade mínima de erro tipo I de 5% (a = 0,05) e uma probabilidade de erro tipo II de 20% (b = 0,2). Esse cálculo foi realizado utilizando-se o software Computer Programs for Epidemiologists (PEPI - Versão 4.04x)

Os animais foram divididos em quatro grupos experimentais (N=7/grupo): controle (CO), monocrotalina (MCT), óleo de copaíba (O), e monocrotalina + óleo de copaíba (MCT-O). No primeiro dia, a HAP foi induzida por uma injeção única de MCT *in bolus* (60mg/kg i.p.), conforme descrito em outro local.^10^ Uma semana depois da indução de HAP, os animais no grupo O e MCT-O receberam óleo de copaíba (400mg/kg) por gavagem, uma vez ao dia, durante 14 dias.^5^ Essa dose corresponde a um volume de 0,63 mL/kg de óleo de copaíba. Durante esse período, os animais dos grupos CO e MCT receberam o mesmo volume de água por gavagem.

### Análise ecocardiográfica

O fluxo pela artéria pulmonar e a função contrátil do VD foram avaliados por ecocardiografia após o final do tratamento para estimar o efeito do óleo de copaíba na função cardiovascular. Os animais foram anestesiados (cetamina, 90 mg/kg; xilazina, 20 mg/kg, intraperitoneal) e colocados na posição decúbito lateral esquerdo para se obter as imagens cardíacas. Foi utilizado um sistema EnVisor Philips (Andover, MA, EUA), com um transdutor de 12–13MHz, por um operador treinado e com experiência em ecocardiografia de pequenos animais. Foram medidos a fração de encurtamento do VD (FEVD), que estima a função contrátil do VD, e o tempo de aceleração (TA) e o tempo de ejeção (TE) dos traços da velocidade do fluxo da artéria pulmonar, que estimam a resistência vascular pulmonar.^17^

### Análise morfométrica

Depois do final do tratamento, os ratos foram sacrificados por decapitação. O VD e o VE foram retirados para medição morfométrica, e foi coletado sangue do tronco venoso para análise de stress oxidativo. VD e VE + septo (S) foram pesados para determinar a hipertrofia cardíaca pelo Índice de Fulton (peso do VD/peso de VE+S).^18^

### Preparação da amostra de sangue:

Defesas antioxidantes sistêmicas e espécies reativas do oxigênio (ERO) totais foram avaliadas nas hemácias e as carbonilas forma medidas no plasma. Amostras de sangue heparinizadas foram lavadas três vezes em uma solução de cloreto de sódio (9g/L) e centrifugadas a 3.000 g por 10 minutos em temperatura ambiente. Eritrócitos lavados foram diluídos na proporção de 1/10 em uma solução de ácido acético (1mmol/L) e sulfato de magnésio (4mmol/L). A solução final foi centrifugada a 4.200 g por 20 minutos. O sobrenadante foi armazenado em freezer a -80 °C para medições de stress oxidativo futuras.^15^

### Concentração de proteína

A concentração de proteína foi quantificada pelo método estabelecido por Lowry et al.,^19^ utilizando albumina bovina como solução padrão na concentração de 1mg/mL. Todos os resultados de stress oxidativo foram normalizados pela quantidade de proteína.^19^

### Determinação dos níveis totais de ERO

A geração de ERO foi medida por emissão de fluorescência DCFH-DA (Sigma-Aldrich, USA). O diacetato de diclorofluoresceína é uma membrana permeável e é rapidamente oxidada para a altamente fluorescente 2’, 7’-diclorofluoresceína (DCF) na presença de ERO. A amostra foi excitada a 488 nm, e a emissão foi coletada com um filtro de passagem longa de 525 nm. As ERO foram expressas como nmol por miligrama de proteína.^20^

### Ensaio de carbonila

Essa técnica se baseia na reação de proteínas oxidadas com 2,4-dinitrofenil-hidrazina (DNPH). Em resumo, estas proteínas foram adicionadas a DNPH 10mmol/L em 2,5mol/L solução de HCl, por 1 h, no escuro em temperatura ambiente, com agitação a cada 15 minutos. Em seguida, uma solução de ácido tricloroacético a 20% (p/v) foi adicionada às amostras de plasma, que foram centrifugadas (1.000 por 5 minutos) para coletar precipitados de proteína. Daí em diante, a pelota foi dissolvida com etanol:acetato de etila (1:1) (v/v) e incubada por 10 minutos a 37 °C com 6mol/L de solução de cloreto de guanidina. A absorbância foi medida em um espectrofotômetro a 360nm e os resultados foram expressos em nmol de DNPH derivativos/mg de proteína.^21^

### Determinação de atividades de enzimas antioxidantes:

A atividade de superóxido dismutase (SOD) foi determinada pela medição da velocidade da formação de pirogalol oxidado, e expressa em unidades por miligrama de proteína, conforme Marklund.^22^ A atividade de catalase (CAT) foi determinada acompanhando a diminuição na absorção de peróxido de hidrogênio de 240 nm. Ela foi expressa em nmol de peróxido de hidrogênio reduzido por minuto por miligrama de proteína.^23^

A atividade de glutationa peroxidase (GPx), expressa em nmol de peróxido de hidrogênio por minuto por mg de proteína, foi medida após a oxidação de NADPH a 340 nm em um meio de reação contendo 0,17 mmol/L de glutationa reduzida, 0,2 U/mL de glutationa redutase, e 0,5 mmol/L de hidroperóxido de terc-butil.^24^

### Níveis totais de glutationa

Os níveis totais de glutationa (GSH) foram determinados conforme descrito por Akerboom e Sies,^25^ com modificações. O GSH foi medido nos eritrócitos após a precipitação de proteína com 10% de ácido tricloroacético. Uma alíquota da amostra foi adicionada ao tampão de fosfato com 500 μmol/L DTNB. O desenvolvimento de cor, resultante da reação entre DTNB e os tióis, atingiu o máximo em 5 minutos e ficou estável por mais 30 minutos. A absorbância foi medida a 412 nm após 10 minutos. Uma curva padrão de glutationa reduzida foi utilizada para calcular os níveis de GSH nas amostras.^25^

### Análise estatística

Para a análise estatística, foi usado o software SigmaPlot. Os dados são mostrados como média ± desvio padrão. O teste de normalidade (Shapiro-Wilks) foi conduzido para determinar a distribuição de dados. Como nossos resultados apresentaram distribuição normal, a análise estatística foi realizada usando ANOVA de duas vias, seguida do teste Student-Newman-Keuls post hoc. A correlação de Pearson foi utilizada para estudar a associação entre variáveis. P-valores menores que 0,05 foram considerados significativos.

## Resultados

### Avaliações ecocardiográfica

Observou-se uma diminuição significativa no TA no grupo MCT, ao mesmo tempo em que houve um aumento no grupo O, em comparação ao CO. Entretanto, o óleo de copaíba recuperou esse parâmetro para controlar os níveis no grupo MCT-O. Por outro lado, não houve diferenças entre os grupos no parâmetro TE. A FEVD, que estima a função sistólica do VD, diminuiu no grupo MCT, e o óleo de copaíba evitou essa alteração nos animais do grupo MCT-O ( Tabela 1 ).

**Tabela 1:** – Resultados ecocardiográficos e morfométricos

	CO	MCT	O	MCT-O
TA (s)	0,029±0,005	0,018±0,001^a^	0,034±0,001^a^	0,025±0,003^b^
TE (s)	0,099±0,01	0,106±0,007	0,098±0,01	0,11±0,001
FEVD (%)	21,2±2,4	9,4±3,1^a^	18,8±2,4	17,8±8,2^b^
Índice de Fulton (mg/mg)	0,29±0,02	0,49±0,01^a^	0,29±0,01	0,39±0,03^b^

*Os dados são expressos como média ± DP. a p <0,05 vs. CO; b p <0,05 vs. MCT. CO: controle; O: óleo de copaíba; MCT: monocrotalina; MCT-O: monocrotalina + óleo; TA: tempo de aceleração; TE: tempo de ejeção; VD: ventrículo direito; FEVD: fração de encurtamento do ventrículo direito. Índice de Fulton = peso do VD/peso do VE + septo; N= 7 animais por grupo.*

### Avaliação morfométrica

O índice de hipertrofia do ventrículo direito (VD/VE + peso de S), mostrado na Tabela 1 , foi significativamente aumentado no grupo MCT em comparação ao grupo CO. Esse parâmetro diminuiu significativamente nos animais do grupo MCT-O.

### Análise de stress oxidativo

**Figura 1:**
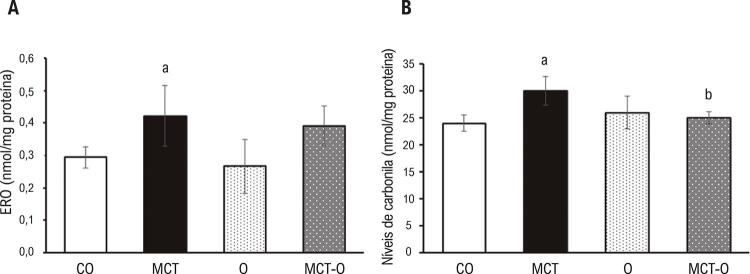
– *Stress oxidativo A) Concentração total de espécies reativas do oxigênio; B) Níveis de carbonila. Os dados são expressos como média ± DP. a p <0,05 vs. CO; b p <0,05 vs. MCT. Grupo de controle: CO; grupo monocrotalina: MCT, grupo óleo de copaíba: O, grupo monocrotalina + óleo de copaíba: MCT-O.*

Houve um aumento significativo nas ERO e nos níveis de carbonila ( Figura 1A e 1B , respectivamente) no grupo MCT em comparação ao CO. Não houve diferença significativa entre o grupo MCT e o MCT-O, ou entre o MCT-O e o O, em relação aos níveis de ERO. Entretanto, os níveis de carbonila, que aumentaram no grupo MCT, foram reduzidos no grupo MCT-O.

### Análise de antioxidantes

**Figura 2:**
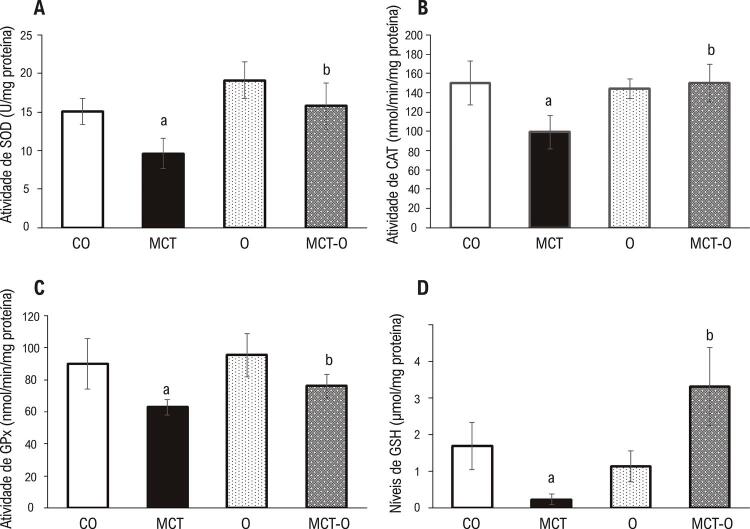
– *Medições de antioxidantes. A) Atividade de superóxido dismutase; B) Atividade de catalase; C) Atividade de glutationa peroxidase; D) Níveis totais de glutationa. Os dados são expressos como média ± DP. a p <0,05 vs. CO; b p <0,05 vs. MCT. Grupo de controle: CO; grupo monocrotalina: MCT, grupo óleo de copaíba: O, grupo monocrotalina + óleo de copaíba: MCT-O*

As atividades de enzimas antioxidantes (SOD, CAT e GPx), e concentração de GSH foram significativamente mais baixas no grupo MCT, em comparação com o grupo CO. O tratamento com óleo de copaíba recuperou esses parâmetros significativamente nos animais do grupo MCT-O. ( Figura 2A, 2B, 2C, 2D , respectivamente).

### Correlações

**Figura 3:**
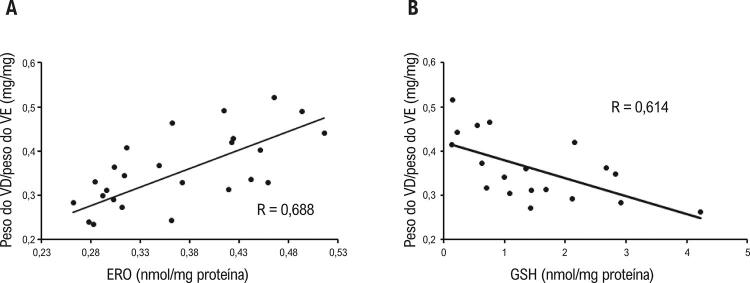
– *Correlações entre peso do VD/peso do VE + S e (A) ERO (P <0,05); e (B) GSH (P<0,05). VD: ventrículo direito, VE: ventrículo esquerdo, ERO: espécies reativas de oxigênio, GSH: níveis totais de glutationa.*

Observou-se uma correlação positiva (R=0,688; p<0,05) entre ERO total e a relação peso de VD/ peso de VE + S ( Figura 3A ), em comparação com uma correlação negativa (R=0,614; p<0,05) entre a concentração de GSH e a relação entre peso de VD/peso de VE + S ( Figura 3B ).

## Discussão

Os principais achados do presente estudo foram que o MCT modulado por óleo de copaíba induziu o *Cor pulmonale* , uma vez que promoveu a redução da resistência arterial pulmonar, além de resultar em disfunção e hipertrofia do VD. Paralelamente a essas alterações, identificou-se que o tratamento com o óleo de copaíba reverteu o stress oxidativo sistêmico aumentado pelo MCT, que foi observado pela melhoria dos níveis de ERO e carbonila, e pela redução das defesas antioxidantes.

De acordo com a literatura, a elevação da resistência pulmonar arterial é acompanhada de hipertrofia do VD, o que caracteriza o *Cor pulmonale.* ^10^ Na verdade, neste estudo observou-se uma diminuição do tempo de aceleração pela artéria pulmonar (TA), o que indica um aumento da resistência da artéria pulmonar, e um aumento da hipertrofia do VD em animais do grupo MCT. Por outro lado, o tratamento com óleo de copaíba conseguiu mitigar esses efeitos. A redução da resistência na artéria pulmonar que contribuiu para o aumento na remodelagem do VD poderia contribuir para melhorar a função do VD. Investigações anteriores em pacientes com HAP indicam que uma deficiência na função do VD está relacionada a resultados clínicos adversos e sobrevida reduzida,^26^ o que destaca a importância de um tratamento, tais como o óleo de copaíba, que ameniza o dano ao VD. Além disso, detectou-se uma diminuição da fração de encurtamento do VD (FEVD) no grupo MCT, o que indica deficiência da função contrátil do VD. Esse resultado está em conformidade com outros,^27 , 28^ que estudaram essa doença. Em contrapartida, conforme descrito no trabalho anterior, o óleo de copaíba conseguiu aumentar esse parâmetro, o que indica que este óleo poderia melhorar a função do VD.^6^

Conforme Jim et al.,^29^ há um aumento na produção de ERO sistêmicas em um modelo de ratos de hipertensão pulmonar. Além disso, Mohammadi^30^ também observou uma redução nas atividades de CAT, SOD, GPx, e na concentração de GSH medida no sangue em HAP induzida por MCT. Esses dados estimularam a investigação dos efeitos antioxidantes do óleo de copaíba sobre a HAP. Os dados deste estudo mostraram que o óleo de copaíba reverteu os danos oxidativos elevando as defesas antioxidantes sistêmicas e reduzindo a carbonila a níveis próximos dos observados nos ratos do grupo de controle, sugerindo que esse óleo tenha aumentado a reserva de antioxidante.

Em um estudo prévio deste grupo, foi avaliada a composição do óleo de copaíba.^5^ A análise química desse óleo foi realizada utilizando cromatografia gasosa com espectrometria de massa. Detectou-se que o óleo de copaíba é composto de terpenos com a predominância do β-cariofileno. Esse composto tem atividade antioxidante, levando à redução das ERO devido a seu efeito de sequestro de radicais livres contra ânions superóxidos, ânions hidroxila, e peróxidos lipídicos.^31^

Portanto, é razoável acreditar que os efeitos antioxidantes observados após o tratamento com óleo de copaíba se devem ao β-cariofileno presente nesse óleo. Por outro lado, os efeitos antioxidantes do óleo de copaíba também podem se dever a uma interação entre seus vários componentes. Conforme relatado em um estudo anterior, o óleo de copaíba é composto de uma grande variedade e outros sesquiterpenos e diterpenos, que também têm propriedades antioxidantes.^5^

O stress oxidativo está envolvido no desenvolvimento da hipertrofia cardíaca patológica e em um prognóstico ruim.^32^ A redução da hipertrofia do VD encontrada pelo presente estudo poderia estar associada ao efeito antioxidante do óleo de copaíba. Na verdade, foi encontrada uma correlação positiva entre os níveis de ERO e a hipertrofia cardíaca neste estudo. Por outro lado, níveis aumentados de GSH, um antioxidante não enzimático endógeno, foram correlacionados a índices mais baixos de hipertrofia cardíaca. Portanto, sugere-se que o óleo de copaíba possa proteger o VD contra a hipertrofia cardíaca por suas propriedades antioxidantes. Esse achado tem enorme importância, pois, de acordo com Rosca et al., a hipertrofia do VD está correlacionada a um risco mais alto de morte súbita cardíaca.^33^ Como essa era uma associação entre stress oxidativo sistêmico e alteração morfométrica cardíaca, esses marcadores podem ser um reflexo das alterações no VD promovidas pela HAP.

O presente estudo foi o primeiro a testar o efeito sistêmico do óleo de copaíba em um modelo de *Cor pulmonale* . Alguns marcadores periféricos de stress oxidativo foram avaliados para se detectar mudanças no estado redox sistêmico. Avaliações do sangue periférico são importantes e úteis, pois refletem o estado de saúde geral do organismo. Amostras de sangue são facilmente acessíveis, disponíveis em grande quantidade, e sua coleta é menos invasiva que uma biópsia de tecido, por exemplo. Portanto, a avaliação dos marcadores de stress oxidativo no sangue de pacientes com HAP poderia ser útil para se monitorar o desenvolvimento do *Cor pulmonale* e o tratamento utilizado, como foi realizado com os animais usados no presente estudo.

## Conclusões

Os resultados obtidos sugerem que o óleo de copaíba tem um efeito antioxidante sistêmico interessante, que se reflete na melhoria da função e na morfometria do VD nesse modelo de *Cor pulmonale* . Esses resultados destacam a importância do óleo de copaíba como possível tratamento adjuvante da HAP.

## References

[1] Kanis LA, Prophiro JS, Vieira Eda S, Nascimento MP, Zepon KM, Kulkamp-Guerreiro IC, et al. Larvicidal activity of copaifera sp. (leguminosae) oleoresin microcapsules against aedes aegypti (diptera: Culicidae) larvae. Parasitol Res. 2012;110(3):1173-8. 10.1007/s00436-011-2610-2 21850452

[2] Veiga Junior VF, Rosas EC, Carvalho MV, Henriques MG, Pinto AC. Chemical composition and anti-inflammatory activity of copaiba oils from copaifera cearensis huber ex ducke, copaifera reticulata ducke and copaifera multijuga hayne--a comparative study. J Ethnopharmacol. 2007;112(2):248-54. 10.1016/j.jep.2007.03.005 17446019

[3] Paiva LA, Gurgel LA, Campos AR, Silveira ER, Rao VS. Attenuation of ischemia/reperfusion-induced intestinal injury by oleo-resin from copaifera langsdorffii in rats. Life Sci. 2004;75(16):1979-87. 10.1016/j.lfs.2004.05.011 15306165

[4] Silva JJ, Pompeu DG, Ximenes NC, Duarte AS, Gramosa NV, Carvalho KM, et al. Effects of kaurenoic acid and arginine on random skin flap oxidative stress, inflammation, and cytokines in rats. Aesthetic Plast Surg. 2015;39(6):971-7. 10.1007/s00266-015-0559-8 26408387

[5] Campos C, de Castro AL, Tavares AM, Fernandes RO, Ortiz VD, Barboza TE, et al. Effect of free and nanoencapsulated copaiba oil on monocrotaline-induced pulmonary arterial hypertension. J Cardiovasc Pharmacol. 2017;69(2):79-85. 10.1097/FJC.0000000000000442 27798416

[6] Carraro CC, Turck P, Seolin BG, Tavares AM, Lacerda D, Corssac GB, et al. Copaiba oil attenuates right ventricular remodeling by decreasing myocardial apoptotic signaling in monocrotaline-induced rats. J Cardiovasc Pharmacol. 2018;72(5):214-21. 10.1097/FJC.0000000000000617 30212415

[7] Chin KM, Rubin LJ. Pulmonary arterial hypertension. J Am Coll Cardiol. 2008;51(16):1527-38. 10.1016/j.jacc.2008.01.024 18420094

[8] Hessel MH, Steendijk P, Adel B, Schutte CI, van der Laarse A. Characterization of right ventricular function after monocrotaline-induced pulmonary hypertension in the intact rat. Am J Physiol Heart Circ Physiol. 2006;291(5):H2424-30. 10.1152/ajpheart.00369.2006 16731643

[9] Maruyama H, Watanabe S, Kimura T, Liang J, Nagasawa T, Onodera M, et al. Granulocyte colony-stimulating factor prevents progression of monocrotaline-induced pulmonary arterial hypertension in rats. Circ J. 2007;71(1):138-43. 10.1253/circj.71.138 17186992

[10] Farahmand F, Hill MF, Singal PK. Antioxidant and oxidative stress changes in experimental cor pulmonale. Mol Cell Biochem. 2004;260(1-2):21-9. 10.1023/b:mcbi.0000026047.48534.50 15228082

[11] Lacerda DS, Turck P, Lima-Seolin B, Colombo R, Ortiz V, Bonetto JH, et al. Pterostilbene reduces oxidative stress, prevents hypertrophy and preserves systolic function of right ventricle in cor pulmonale model. Br J Pharmacol. 2017;174(19):3302-14. 10.1111/bph.13948 PMC5595755 28703274

[12] Fessel JP, West JD. Redox biology in pulmonary arterial hypertension (2013 grover conference series). Pulm Circ. 2015;5(4):599-609. 10.1086/683814 PMC4671734 26697167

[13] Tabima DM, Frizzell S, Gladwin MT. Reactive oxygen and nitrogen species in pulmonary hypertension. Free Radic Biol Med. 2012;52(9):1970-86. 10.1016/j.freeradbiomed.2012.02.041 PMC3856647 22401856

[14] Grobe AC, Wells SM, Benavidez E, Oishi P, Azakie A, Fineman JR, et al. Increased oxidative stress in lambs with increased pulmonary blood flow and pulmonary hypertension: Role of nadph oxidase and endothelial no synthase. Am J Physiol Lung Cell Mol Physiol. 2006;290(6):L1069-77. 10.1152/ajplung.00408.2005 16684951

[15] Repetto MG, Reides CG, Evelson P, Kohan S, de Lustig ES, Llesuy SF. Peripheral markers of oxidative stress in probable alzheimer patients. Eur J Clin Invest. 1999;29(7):643-9. 10.1046/j.1365-2362.1999.00506.x 10411672

[16] Fois AG, Paliogiannis P, Sotgia S, Mangoni AA, Zinellu E, Pirina P, et al. Evaluation of oxidative stress biomarkers in idiopathic pulmonary fibrosis and therapeutic applications: A systematic review. Respir Res. 2018;19(1):51. 10.1186/s12931-018-0754-7 PMC5872514 29587761

[17] Urboniene D, Haber I, Fang YH, Thenappan T, Archer SL. Validation of high-resolution echocardiography and magnetic resonance imaging vs. High-fidelity catheterization in experimental pulmonary hypertension. Am J Physiol Lung Cell Mol Physiol. 2010;299(3):L401-12. 10.1152/ajplung.00114.2010 PMC2951068 20581101

[18] Winter RL, Ray Dillon A, Cattley RC, Blagburn BL, Michael Tillson D, Johnson CM, et al. Effect of heartworm disease and heartworm-associated respiratory disease (hard) on the right ventricle of cats. Parasit Vectors. 2017;10(Suppl 2):492. 10.1186/s13071-017-2451-7 PMC5688429 29143659

[19] Lowry OH, Rosebrough NJ, Farr AL, Randall RJ. Protein measurement with the folin phenol reagent. J Biol Chem. 1951;193(1):265-75. 14907713

[20] LeBel CP, Ischiropoulos H, Bondy SC. Evaluation of the probe 2’,7’-dichlorofluorescin as an indicator of reactive oxygen species formation and oxidative stress. Chem Res Toxicol. 1992;5(2):227-31. 10.1021/tx00026a012 1322737

[21] Reznick AZ, Packer L. Oxidative damage to proteins: Spectrophotometric method for carbonyl assay. Methods Enzymol. 1994;233:357-363. 10.1016/s0076-6879(94)33041-7 8015470

[22] Marklund SL. Superoxide dismutase isoenzymes in tissues and plasma from new zealand black mice, nude mice and normal balb/c mice. Mutat Res. 1985;148(1-2):129-34. 10.1016/0027-5107(85)90216-7 3969077

[23] Boveris A, Chance B. The mitochondrial generation of hydrogen peroxide. General properties and effect of hyperbaric oxygen. Biochem J. 1973;134(3):707-16. 10.1042/bj1340707 PMC1177867 4749271

[24] Flohe L, Gunzler WA. Assays of glutathione peroxidase. Methods Enzymol. 1984;105:114-21. 10.1016/s0076-6879(84)05015-1 6727659

[25] Akerboom TP, Sies H. Assay of glutathione, glutathione disulfide, and glutathione mixed disulfides in biological samples. Methods Enzymol. 1981;77:373-82. 10.1016/s0076-6879(81)77050-2 7329314

[26] Vanderpool RR, Pinsky MR, Naeije R, Deible C, Kosaraju V, Bunner C, et al. Rv-pulmonary arterial coupling predicts outcome in patients referred for pulmonary hypertension. Heart. 2015;101(1):37-43. 10.1136/heartjnl-2014-306142 PMC4268056 25214501

[27] Turck P, Lacerda DS, Carraro CC, de Lima-Seolin BG, Teixeira RB, Bonetto JH, et al. Trapidil improves hemodynamic, echocardiographic and redox state parameters of right ventricle in monocrotaline-induced pulmonary arterial hypertension model. Biomed Pharmacother. 2018;103:182-90. 10.1016/j.biopha.2018.04.001 29653363

[28] Ruiter G, de Man FS, Schalij I, Sairras S, Grunberg K, Westerhof N, et al. Reversibility of the monocrotaline pulmonary hypertension rat model. Eur Respir J. 2013;42(2):553-6. 10.1183/09031936.00012313 23904554

[29] Jin H, Liu M, Zhang X, Pan J, Han J, Wang Y, et al. Grape seed procyanidin extract attenuates hypoxic pulmonary hypertension by inhibiting oxidative stress and pulmonary arterial smooth muscle cells proliferation. J Nutr Biochem. 2016;36:81-8. 10.1016/j.jnutbio.2016.07.006 27596528

[30] Mohammadi S, Najafi M, Hamzeiy H, Maleki-Dizaji N, Pezeshkian M, Sadeghi-Bazargani H, et al. Protective effects of methylsulfonylmethane on hemodynamics and oxidative stress in monocrotaline-induced pulmonary hypertensive rats. Adv Pharmacol Sci. 2012;2012:507278. 10.1155/2012/507278 PMC3478703 23118745

[31] Calleja MA, Vieites JM, Montero-Melendez T, Torres MI, Faus MJ, Gil A, et al. The antioxidant effect of beta-caryophyllene protects rat liver from carbon tetrachloride-induced fibrosis by inhibiting hepatic stellate cell activation. Br J Nutr. 2013;109(3):394-401. 10.1017/S0007114512001298 22717234

[32] Madamanchi NR, Runge MS. Redox signaling in cardiovascular health and disease. Free Radic Biol Med. 2013;61:473-501. 10.1016/j.freeradbiomed.2013.04.001 PMC3883979 23583330

[33] Rosca M, Calin A, Beladan CC, Enache R, Mateescu AD, Gurzun MM, et al. Right ventricular remodeling, its correlates, and its clinical impact in hypertrophic cardiomyopathy. J Am Soc Echocardiogr. 2015;28(11):1329-38. 10.1016/j.echo.2015.07.015 26296986

