# Supplementary material for: Efeitos do Óleo de Copaíba em Marcadores Periféricos de Estresse Oxidativo em um Modelo de Cor Pulmonale em Ratos
Source: Arq Bras Cardiol. 2021 Sep 16;117(6):1106–12. [Article in Portuguese] doi: 10.36660/abc.20200929 (PMC8757149; doi:10.36660/abc.20200929)
Supplement: Translation

# Translation: Effects of Copaiba Oil in Peripheral Markers of Oxidative Stress in a Model of Cor Pulmonale in Rats

**Authors:** Cristina Campos, Patrick Turck, Angela Maria Vicente Tavares, Giana Corssac, Denise Lacerda, Alex Araujo, Susana Llesuy, Adriane Bello Klein

**Affiliations:** 1 Brazil Universidade Federal do Rio Grande do Sul, Porto Alegre, RS – Brazil; 2 Argentina Hospital Italiano de Buenos Aires, Buenos Aires – Argentina

**Keywords:** Cor Pulmonale, Monocrotaline,Rats, Oxidative Stress, Fabaceae, Phytotherapy, Hypertrophy, Right Ventricular, Copaiba Oil

## Abstract

**Background:**

To date, copaiba oil’s systemic effects have never documented in Cor pulmonale induced by monocrotaline.

**Objectives:**

To investigate copaiba oil’s effects in peripheral markers of oxidative stress in rats with Cor pulmonale.

**Methods:**

Male Wistar rats (170±20g, n=7/group) were divided into four groups: control (CO), monocrotaline (MCT), copaiba oil (O), and monocrotaline+copaiba oil (MCT-O). MCT (60 mg/kg i.p.) was administered, and after one week, treatment with copaiba oil (400 mg/kg/day-gavage-14 days) was begun. Echocardiography was performed and, later, trunk blood collection was performed for oxidative stress evaluations. Statistical analysis: two-way ANOVA with Student-Newman-Keuls post-hoc test. P values<0.05 were considered significant.

**Results:**

Copaiba oil reduced pulmonary vascular resistance and right ventricle (RV) hypertrophy (Fulton index (mg/mg): MCT-O=0.39±0.03; MCT=0.49±0.01), and improved RV systolic function (RV shortening fraction, %) in the MCT-O group (17.8±8.2) as compared to the MCT group (9.4±3.1; p<0.05). Moreover, in the MCT-O group, reactive oxygen species and carbonyl levels were reduced, and antioxidant parameters were increased in the peripheral blood (p<0.05). Conclusions: Our results suggest that copaiba oil has an interesting systemic antioxidant effect, which is reflected in the improvements in function and RV morphometry in this Cor pulmonale model. Cor pulmonale attenuation promoted by copaiba oil coincided with a reduction in systemic oxidative stress.

## Introduction

The Amazon forest could be considered a natural laboratory, since it has a wide diversity of plants with medicinal properties. The great majority of these plants have not yet been fully studied, as it is the case of copaiba.^1^ Copaiba is a large tree that grows abundantly in the northern region of Brazil. Since the 16th century, copaiba oil has been used by the native indigenous people of the country in the treatment of various diseases. These traditional uses have motivated some researchers to study this oil.^2^

According to some reports, copaiba oil presents antioxidant and anti-lipoperoxidative properties.^3 , 4^ The antioxidant properties of copaiba oil could be very useful in the treatment of some cardiovascular diseases associated with oxidative stress. However, only two studies were found in the literature demonstrating the beneficial effects of copaiba oil on cardiovascular diseases, such as pulmonary arterial hypertension (PAH).^5 , 6^

PAH is a chronic and fatal disease that is associated with progressive increases in pulmonary vascular resistance and pressure. These changes impair the performance of the right ventricle (RV) and result in RV failure, and ultimately death.^7^ To study the physiopathological mechanisms involved in RV dysfunction and PAH development, a monocrotaline (MCT) model was used.^8^ The active metabolite of MCT causes damage in the pulmonary endothelium, leading to PAH.^9^

The MCT model mimics aspects of human PAH, including *Cor pulmonale,* which is a term used to describe pathological RV hypertrophy induced by lung dysfunction.^10^ In fact, multiple studies in animal models and patients implicate oxidative stress in the development of *Cor pulmonale* and PAH.^11 - 13^ Oxidative stress can cause damage to pulmonary endothelial cells,^14^ as well as contribute to RV dysfunction and failure.^11^ However, no study has explored the impact of PAH on oxidative stress markers in peripheral blood by analyzing copaiba oil’s effects. It was reported that oxidative stress measured in the blood of patients with a neurodegenerative disease could represent a reflection of the oxidative brain damage in those patients.^15^ In this sense, evaluating peripheral markers of oxidative stress could have clinical applicability, since obtaining a blood sample represents a minimally invasive procedure.^16^ This approach could be useful to cardiopulmonary diseases, such as PAH, in order to monitor the disease’s progression, together with the need and efficacy of antioxidant therapy, such as copaiba oil.

Thus, the aim of this study was to investigate whether peripheral markers of oxidative stress reflect the structural and functional changes promoted in RV by PAH and the effects of copaiba oil under these markers.

## Methods

### Animals, induction of cor pulmonale, and groups:

All procedures were approved by the institutional Animal Ethics Committee (protocol number: 31765). In total, 28 male Wistar rats (weighing 170±20g) from the Center for Reproduction and Experimentation of Laboratory Animals (CREAL) of Universidade Federal do Rio Grande do Sul were studied. These were kept at 20-22°C and with a 12:12h dark/light photoperiod. All animals had *ad libitum* access to regular rodent chow and water, and the experiments were conducted in accordance with the Guide for the Care and Use of Laboratory Animals (U.S. Department of Health and Human Services, NIH Publication No. 86-23) and with institutional guidelines.

The number of animals per group was estimated based on previous studies from our research group,^6 , 7^ considering the minimum difference between the groups of two standard deviations, a minimum probability of type I error of 5% (a = 0.05), and a probability of type II error of 20% (b = 0.2). This calculation was performed using the Computer Programs for Epidemiologists (PEPI - Version 4.04x) software.

Animals were divided into four experimental groups (N=7/group): control (CO), copaiba oil (O), monocrotaline (MCT), and monocrotaline+copaiba oil (MCT-O). On the first day, PAH was induced by a single *in bolus* MCT injection (60mg/kg i.p.), as described elsewhere.^10^ One week after PAH induction, animals in the O and MCT-O groups received copaiba oil (400mg/kg) by gavage, once a day, for 14 days.^5^ This dose corresponds to a volume of 0.63mL/kg of copaiba oil. During this period, animals from the CO and MCT groups received the same volume of water by gavage.

### Echocardiographic analysis

The flow through the pulmonary artery and RV contractile function were evaluated by echocardiography after the end of treatment to estimate the effect of copaiba oil on the cardiovascular function. Animals were anesthetized (ketamine, 90mg/kg; xylazine, 20mg/kg, intraperitoneal) and placed in the left lateral decubitus position to obtain cardiac images. An EnVisor Philips system (Andover, MA) was used, with a 12–13MHz transducer, by a trained operator with experience in small animal echocardiography. The RV shortening fraction (RVSF), which estimates RV contractile function, as well as the acceleration time (AT) and the ejection time (ET) of pulmonary artery flow velocity tracings were measured, which estimates pulmonary vascular resistance.^17^

### Morphometric analysis

After the end of treatment, rats were killed by decapitation. RV and left ventricle (LV) were harvested for morphometric measurement, and blood was collected from the venous trunk for oxidative stress analysis. RV and LV+septum (S) were weighed to determine cardiac hypertrophy through the Fulton index (RV weight/LV+S weight).^18^

### Blood sample preparation:

Systemic antioxidant defenses and total reactive oxygen species (ROS) were evaluated in red blood cells, and carbonyls were measured in plasma. Heparinized blood samples were washed three times in a solution of sodium chloride (9g/L) and centrifuged at 3,000g for 10min. at room temperature. Washed erythrocytes were diluted 1/10 in a solution of acetic acid (1mmol/L) and magnesium sulphate (4mmol/L). The final solution was centrifuged at 4,200g for 20 min. The supernatant was stored in freezers at -80ºC for later oxidative stress measurements.^15^

### Protein concentration

Protein concentration was quantified by the method established by Lowry et al.,^19^ using bovine albumin as a standard solution at a concentration of 1mg/mL. All oxidative stress results were normalized by the amount of protein.^19^

### Determination of total ROS levels

ROS generation was measured by DCFH-DA fluorescence emission (Sigma-Aldrich, USA). Dichlorofluorescein diacetate is a permeable membrane and is rapidly oxidized to the highly fluorescent 2,7-dichlorofluorescein (DCF) in the presence of ROS. The samples were excited at 488 nm and emission was collected with a 525nm long pass filter. ROS was expressed as nmol per milligram of protein.^20^

### Carbonyl assay

This technique is based on the reaction of oxidized proteins with 2,4-dinitro-phenyl hydrazine (DNPH). Briefly, these proteins were added to a DNPH 10mmol/L in 2.5mol/L HCl solution for 1 h in the dark at room temperature, with shaking every 15 min. A 20% trichloroacetic acid (w/v) solution was then added to the plasma samples, which were centrifuged (1,000g for 5 min) to collect protein precipitates. Thereafter, the pellet was dissolved with ethanol:ethyl acetate (1:1) (v/v) and incubated for 10 min at 37ºC with 6mol/L guanidine hydrochloride solution. The absorbance was measured in a spectrophotometer at 360nm and results were expressed as nmol DNPH derivatives/mg protein.^21^

### Determination of antioxidant enzyme activities:

Superoxide dismutase (SOD) activity was determined by measuring the velocity of oxidized pyrogalol formation, and expressed as units per milligram of protein, according to Marklund.^22^ Catalase (CAT) activity was determined by following the decrease in 240nm absorption of hydrogen peroxide. This was expressed as ɳmol of hydrogen peroxide reduced per minute per milligram of protein.^23^

Glutathione peroxidase (GPx) activity, expressed as nmol of hydroperoxide reduced per min per mg protein, was measured following NADPH oxidation at 340nm in a reaction medium containing 0.17mmol/L reduced glutathione, 0.2U/mL glutathione reductase, and 0.5mmol/L tert-butyl hydroperoxide.^24^

### Total glutathione levels

Total glutathione (GSH) levels were determined as described by Akerboom and Sies,^25^ with modifications. GSH was measured in erythrocytes after protein precipitation with 10% trichloroacetic acid. An aliquot of the sample was added to phosphate buffer with 500μmol/L DTNB. Color development, resulting from the reaction between DTNB and the thiols, reached a maximum in 5 min and was stable for more than 30 min. Absorbance was read at 412nm after 10 min. A standard curve of reduced glutathione was used to calculate the GSH levels in the samples.^25^

### Statistical Analysis

For statistical analysis, the SigmaPlot software was used. Data are shown as mean±standard deviation. The normality test (Shapiro-Wilks) was performed to determine the data distribution. As our results presented normal distribution, statistical analysis was performed using two-way ANOVA, followed by the Student-Newman-Keuls post-hoc test. Pearson correlation was used to study the association between variables. P values less than 0.05 were considered significant.

## Results

### Echocardiographic evaluations

A significant decrease was observed in the AT in the MCT group, while it was increased in the O group, as compared to CO. However, copaiba oil recovered this parameter to control levels in the MCT-O group. By contrast, no difference was found among groups in the ET parameter. RVSF, which estimates RV systolic function, decreased in the MCT group, and copaiba oil prevented this change in the MCT-O animals ( Table 1 ).

**Table 1:** – Echocardiographic and morphometric results

	CO	MCT	O	MCT-O
AT (s)	0.029±0.005	0.018±0.001^a^	0.034±0.001^a^	0.025±0.003^b^
ET (s)	0.099±0.01	0.106±0.007	0.098±0.01	0.11±0.001
RVSF (%)	21.2±2.4	9.4±3.1^a^	18.8±2.4	17.8±8.2^b^
Fulton index (mg/mg)	0.29±0.02	0.49±0.01^a^	0.29±0.01	0.39±0.03^b^

*Data are expressed as mean±SD. a P<0.05 vs CO; b P<0.05 vs MCT. CO:control; O: Copaiba oil; MCT: Monocrotaline; MCT-O: Monocrotaline+Oil; AT: acceleration time; ET: ejection time; RV: right ventricle; RVSF: right ventricle shortening fraction. Fulton index: RV weight/LV+Septum weight; N: 7 animals per group.*

### Morphometric evaluation

The right ventricular hypertrophy index (RV/LV+S weight), shown in Table 1 , was significantly increased in the MCT group in comparison to the CO group. This parameter decreased significantly in the animals from the MCT-O group.

### Oxidative stress analysis

There was a significant increase in the ROS and carbonyl levels ( Figure 1A and 1B , respectively) in the MCT group in comparison with CO. No significant difference was found between the MCT group and the MCT-O, or between the MCT-O and the O group in ROS levels. However, carbonyl levels, which increased in the MCT group, were reduced in MCT-O.

**Figure 1:** – *Oxidative stress A) Total reactive oxygen species concentration; B) Carbonyl levels. Data are expressed as mean±SD. a P<0.05 vs CO; b P<0.05 vs MCT. Control group: CO; Monocrotaline group: MCT, Copaiba oil group: O, Monocrotaline+Copaiba oil group: MCT-O.*

### Antioxidant analysis

Antioxidant enzyme activities (SOD, CAT, and GPx), and GSH concentration were significantly lower in the MCT group, when compared to the CO group. Treatment with copaiba oil significantly recovered these parameters in the animals from the MCT-O group ( Figure 2A, 2B, 2C, 2D , respectively).

**Figure 2:** – *Antioxidant measurements. A) Superoxide dismutase activity; B) Catalase activity; C) Glutathione peroxidase activity; D) Total glutathione levels. Data are expressed as mean±SD. a P<0.05 vs CO; b P<0.05 vs MCT. Control group: CO; Monocrotaline group: MCT; Copaiba oil group: O; Monocrotaline+Copaiba oil group: MCT-O.*

### Correlations

A positive correlation (R=0.688; p<0.05) was observed between total ROS and the RV weight/LV+S weight ratio ( Figure 3A ), as compared to a negative correlation (R=0.614; p<0.05) between GSH concentration and the RV weight/LV+S weight ratio ( Figure 3B ).

**Figure 3:** – *Correlations between RV weight/LV+S weight and (A) ROS (p < 0.05); and (B) GSH (p<0.05). RV: right ventricle; LV: left ventricle; ROS: reactive oxygen species; GSH:Total glutathione levels.*

## Discussion

The main findings of the present study where that copaiba oil modulated MCT-induced *Cor pulmonale* , since it promoted reduction in the pulmonary artery resistance, as well as in RV hypertrophy and dysfunction. In parallel with these changes, it was found that: copaiba oil treatment reversed the systemic oxidative stress increased by MCT, which was observed by enhanced ROS and carbonyl levels, and a reduction in antioxidant defenses.

According to the literature, the elevation in the pulmonary artery resistance is accompanied by RV hypertrophy, which characterizes *Cor pulmonale.* ^10^ In fact, in this study, a decrease in the acceleration time through pulmonary artery (AT) was observed, which indicates an increase in pulmonary artery resistance and an increase in RV hypertrophy in animals from the MCT group. On the other hand, copaiba oil treatment was able to mitigate these effects. The reduction of resistance in the pulmonary artery, which contributed to an improvement in RV remodeling, could contribute to improving the RV function. Previous investigations in patients with PAH indicate that a RV function impairment is related to adverse clinical outcomes and reduced survival,^26^ which highlights the importance of a treatment, such as copaiba oil, which softens this harmful damage in the RV. Moreover, a decrease was found in the RV shortening fraction (RVSF) in the MCT group, which indicates impairment of the RV contractile function. This result is in accordance with others^27 , 28^ that have studied this disease. Conversely, as described in a previous work, copaiba oil was able to increase this parameter, which indicates that this oil could improve the RV function.^6^

According to Jim et al.,^29^ there is an increase in systemic ROS production in a rat model of pulmonary hypertension. Moreover, Mohammadi^30^ also observed a decrease in the CAT, SOD, GPx activities, and GSH concentration measured in the blood in an MCT-induced PAH. Those data encouraged us to investigate the antioxidant effects of copaiba oil on PAH. Our data have shown that copaiba oil reversed oxidative damage by elevating the systemic antioxidant defenses and reducing carbonyl to levels close to that observed in control rats, suggesting that this oil has enlarged the antioxidant reserve.

In a previous study from our group, copaiba oil composition was evaluated.^5^ Chemical analysis of this oil was carried out using gas chromatography – mass spectroscopy. It was found that copaiba oil is composed of terpenes with the predominance of β-caryophyllene. This compound has antioxidant activity, leading to a reduction in ROS due to its free radical-scavenging effect against superoxide anions, hydroxyl anions, and lipid peroxides.^31^

Thus, it is reasonable to believe that the antioxidant effects observed after treatment with copaiba oil are due to the β-caryophyllene present in this oil. On the other hand, the antioxidant effects of copaiba oil may also be due to an interaction between its various components. As reported in a previous study, copaiba oil is composed of a large variety of other sesquiterpenes and diterpenes, which also have antioxidant properties.^5^

Oxidative stress is involved in the development of pathologic cardiac hypertrophy and in a bad prognosis.^32^ The reduction in RV hypertrophy found in the present study could be associated with the copaiba oil antioxidant effect. In fact, a positive correlation was found between ROS levels and cardiac hypertrophy in this study. On the other hand, increased levels of GSH, an endogenous non-enzymatic antioxidant, were correlated with lower rates of cardiac hypertrophy. Thus, it is suggested that copaiba oil can protect RV against cardiac hypertrophy through its antioxidant proprieties. This finding is of tremendous importance because, according to Rosca et al., RV hypertrophy is correlated with an increased risk of sudden cardiac death.^33^ Since there was an association between systemic oxidative stress and a cardiac morphometric alteration, these markers may be a reflection of the changes in the RV promoted by PAH.

The present study was the first to test the systemic effect of copaiba oil in a *Cor pulmonale* model. Some oxidative stress peripheral markers to detect changes in the systemic redox state were evaluated. Evaluations in the peripheral blood are important and useful because they reflect the organism’s general state of health. Blood samples are easily accessible, available in large quantity, and its collection is less invasive than a tissue biopsy, for example. Thus, the evaluation of oxidative stress markers in the blood of patients with PAH could be useful to monitor the development of *Cor pulmonale* and the applied treatment, as performed in the animals used in the present study.

## Conclusions

The results obtained suggest that copaiba oil has an interesting systemic antioxidant effect, which is reflected in the RV morphometric and function improvement in this *Cor pulmonale* model. These results highlight the importance of copaiba oil as a potential adjuvant treatment for PAH.
